# Parent Attitudes regarding Orthodontists' Role as Potential Administrators of Human Papilloma Virus (HPV) Vaccines

**DOI:** 10.1155/2022/6541532

**Published:** 2022-06-06

**Authors:** Gloria Lee, Jessica Begley, Kavita Ahluwalia, Jaffer A Shariff, Sunil Wadhwa, Christine O'Hea

**Affiliations:** ^1^Private Practice, WI, USA; ^2^Department of Orthodontics, University of Illinois at Chicago College of Dentistry, Chicago, IL, USA; ^3^Department of Dental Public Health, College of Dental Medicine, Columbia University, NY, USA; ^4^Department of Periodontics, Touro College of Dental Medicine, New York Medical College, NY, USA; ^5^Department of Orthodontics, College of Dental Medicine, Columbia University, NY, USA

## Abstract

**Objective:**

To assess parent attitudes regarding orthodontists' role as potential administrators of human papilloma virus (HPV) vaccines.

**Materials and Methods:**

275 parents of adolescents, aged 11–17, who attended the orthodontic clinic at an American university for orthodontic adjustment visits and met inclusion criteria were given information about HPV and HPV vaccines. A paper questionnaire was administered to assess comfort level with orthodontists as HPV vaccinators. Demographic and other potential explanatory characteristics were collected. Descriptive, bivariate, and multivariate ordinal logistic regression analyses were performed with SPSS statistical software v25.

**Results:**

The majority of participants were between 31 and 40 years old, with 79.6% identifying as female. 54.3% of the subjects' children identified as female. Although 71.3% of participants identified as Hispanic, 55.3% of the total participants chose to respond to the questionnaire in Spanish. 66.7% of the participants reported education level as high school degree or less. Overall, 52.4% of parents responded that they would be comfortable with orthodontists administering HPV vaccines to their children. Bivariate analysis suggested a significant association (*p* < 0.05) of parents taking the survey in Spanish and parents' educational attainment with HPV vaccine administration comfort level. Multivariate ordinal logistic regression indicates that parents taking survey in Spanish (adjusted OR: 2.42, 95% CI: 1.24–4.72; *p* < .01) and parents of male children (adjusted OR: 1.66, 95% CI: 1.01–2.73; *p* < 047) were comfortable with orthodontists administering the HPV vaccine.

**Conclusions:**

The language of the survey influenced parents' comfort level with orthodontists as HPV vaccinators, with Spanish having a positive correlation to comfort level. Parents of male children were more comfortable with orthodontists as HPV vaccinator.

## 1. Introduction

In the United States, 79 million people, mostly in their late teens or early 20s, are infected with the human papilloma virus (HPV), which is the most common sexually transmitted disease [1]. Every year, 6 million young people are infected with genital HPV infections from sexual activity [2], and approximately 44,000 new HPV related cancers occur in all ages [3]. The particular concern for dental professionals is that HPV can cause Head and Neck Squamous Cell Carcinomas (HNSCC), which can arise in the oral cavity, oropharynx, larynx, or hypopharynx [4]. Oropharyngeal cancers are the sixth most common cancers in the world [5] with 70–80% of those being associated with HPV [3, 6].

There have been over 200 different HPVs identified. These are characterized as low risk or high risk based on their potential to cause malignant HNSCC. High risk HPV strains, such as HPV16 and HPV18, can cause premalignant squamous intraepithelial neoplasia that can progress to cancer [7]. When HPV enters the host cell chromosome and expresses high levels of the viral oncoproteins E6 and E7, critical tumor suppressor proteins (p53 and pRb, respectively) are inactivated. Inactivation of these proteins causes loss of cell cycle control, impaired cell differentiation, increased mutations, and chromosomal instability leading to tumor production [8].

pRb prevents excessive cell growth by inhibiting cell cycle progression until a cell is ready to divide. When the cell is ready to divide, pRb is phosphorylated (inactivated) by cyclin-dependent kinases and the cell cycle can progress. Another tumor suppressor protein, p16, controls pRb phosphorylation by inhibiting cyclin-dependent kinases. The HPV viral oncoprotein, E7, inactivates pRb which causes an overexpression of p16 in HPV related tumors in an unsuccessful attempt to stop cell proliferation [9]. Intracellularly, hypermethylation in specific gene promotor regions is a common feature found in cancerous DNA. Ferlazzo et al. found a higher frequency of hypermethylation of p16 in their promotor regions in oral squamous cell cancer (OSCC) patients than in healthy controls [10].

Salivary biomarkers can be used to detect HPV infections and HPV related oral squamous cell cancers. Saliva analysis can be a pain-free, noninvasive option for the diagnosis of OSCC [11]. The presence of the viral oncoproteins E6 and E7 indicate HPV infection whereas high levels of p16 have been found to be a strong indicator of HPV related OSCC [12].

Increases in HPV infections in younger populations (<25 years old) reflect earlier sexual activity, increasing numbers of sexual partners, and orogenital sexual habits in this population [5, 13]. In 2011, approximately 66% of both boys and girls of ages 15–24 were reported to have had oral sex and sexual intercourse [5]. Such trends necessitate education on sexually transmitted infections such as HPV and HPV vaccine recommendation for adolescents in all appropriate settings.

HPV vaccines became first available in 2006. Gardasil 9, a nonavalent HPV vaccine became available in 2014 and was recommended for use by the Advisory Committee on Immunization Practices in 2015 [14, 15]. Routine HPV vaccination is recommended to start or complete by age 11–12, and the first dose can be administered as early as age 9. If HPV vaccination is initiated before age 15, a 2-dose schedule is followed with the doses being six months apart [5, 16]. After age 15, a 3-dose schedule is followed, with one to two months between the first and second doses, and six months between the first and last doses [16]. In both dosing scenarios, multiple visits to a provider's office are necessary.

Despite the HPV vaccine availability, reports in 2018 showed that only about half of adolescents in US had received the vaccination, and about 66% of teenagers aged 13–17 had received the first dose [17, 18]. The low rate of HPV vaccine uptake is largely associated with lack of knowledge or awareness of HPV infection and vaccination. Because greater awareness of HPV leads to greater uptake of HPV vaccination [19, 20], healthcare providers other than primary care physicians would be excellent additional resources to promote and administer HPV vaccines. Dentists, and specifically orthodontists, are well positioned to provide advice regarding HPV and HPV vaccinations because they have frequent contact with patients [21]. HPV is associated with oropharyngeal cancers, but recent data from 2018 suggest that parents generally do not feel comfortable with dentists administrating HPV vaccines [22]. However, in 2020, Dean et al. found that the majority of parents in their study were comfortable with pediatric dentists administering the HPV vaccine to their children [23]. Orthodontists are in an ideal position as key players in HPV prevention because their patient population consists predominately of adolescents, patients are seen at regular monthly intervals, and orthodontists are trusted providers. Thus, orthodontists can play a primary role in the delivery of a multidose series vaccine that requires multiple visits to the provider's office.

Earlier studies have looked at patient/parent characteristics associated with HPV vaccination. Females have been found to be more familiar with HPV infections and vaccines than males [21, 24–26]. Blacks and Hispanics were less likely to have heard of HPV infection and the vaccine than non-Hispanic whites [26]. However, other studies found that Hispanic and other minority adolescents, including Asians, were more likely to be vaccinated for HPV than white adolescents [27]. Mixed results have been reported regarding the association of the education level with the uptake of HPV vaccines.[19]

The purpose of this study is to assess parents' perceptions of orthodontists as potential administrators of the HPV vaccine. The rationale for this study is based on the growing prevalence of preventable HPV related oropharyngeal cancers and public health efforts to expand access to the HPV vaccine. Since the vaccine is targeted at adolescents, parents play a lead role in a child's health decisions. Additionally, parents of children undergoing orthodontic treatment have an excellent opportunity to establish a relationship with the orthodontist as the appointments are frequent and the treatment is normally years in duration. Exploring parent attitudes regarding orthodontists as potential HPV vaccinators can open new opportunities for getting more of the US adolescent population vaccinated against HPV, thereby decreasing the prevalence and incidence of HPV infections and associated cancers. Additionally, the results of this study may give good reasoning for orthodontists playing a future role in administering other vaccines, such as those developed for COVID-19.

## 2. Materials and Methods

Inclusion criteria included parents of patients between the ages of 11 and 17 who had been treated by the same orthodontic provider for at least six months prior to our study, which was indicative of an ongoing relationship between the patient/parent and orthodontic provider. Parent subjects were approached at their child's monthly orthodontic appointment and given information by one of three clinician investigators on the research team. Each clinician read from a script in English or Spanish based on the subject's preference. The subject was given general information about HPV and HPV vaccines as well as the research project that they were being asked to participate in. Subjects were encouraged to ask questions about any aspect of HPV, HPV vaccines, and/or the present study. Once the subject agreed to participate, a paper survey instrument was used to collect sociodemographics, comfort level with orthodontists as administrators of the HPV vaccine, and participant's perceived benefits vs. concerns regarding orthodontists as vaccine administrators. Subjects signed a consent form to participate in the study and filled out the paper survey out in the waiting room. The participants were given the choice of completing the survey in either Spanish or English. Prior vaccination status of the child was not considered.

The survey was a modification of that used in Lazalde et al.'s study, “parent perceptions of dentists' role in HPV vaccination,” [22] in which the term “dentist” used in the original questionnaire was replaced by “orthodontist.” Questions on demographic characteristics of the parent and child, such as their age and sex, relationship with the child, race or ethnicity, and highest education level, were also added to the questionnaire.

Subjects were able to choose 5 responses to the question regarding comfort level of orthodontists as vaccinators: very uncomfortable, somewhat uncomfortable, neither uncomfortable nor comfortable, somewhat comfortable, and very comfortable. Participants were stratified by comfort with orthodontist administration of the HPV vaccine (comfortable, neither comfortable nor uncomfortable, and uncomfortable). These 3 categories were used for all statistical analyses. A Kruskal–Wallis test was used to assess the relationship between key sociodemographic variables and comfort with orthodontists as HPV vaccine administrators. These sociodemographic variables are listed in Table 1. An ordinal logistic regression analysis was also used to determine the association between the aforementioned variables and comfort level. A critical *α* of 0.05 was used to test significance for all analyses, which were conducted using IBM SPSS software v25.

## 3. Results

### 3.1. Sample Characteristics

A convenience sample of 296 participants was approached to participate in the study, but 275 chose to participate resulting in a response rate of 92.9%. Characteristics of the participants are shown in Table 2. The majority of the parents/guardians were female between 30 and 40 years old. 54.3% of the participants' children identified as female with half being between 13 and 15 years old.

196 participants (71.3%) identified as Hispanic with 152 (55.2%) of the total participants choosing to complete the questionnaire in Spanish. 97.4% of the participants who completed the questionnaire in Spanish self-identified as Hispanic.

27.3% of participants had less than a high school education, 38.2% graduated from high school, and 25.5% obtained Bachelor's degree. Only 6.3% of subjects achieved an education level higher than a Bachelor's degree.

### 3.2. Parent Comfort with Orthodontists as HPV Vaccinators

About half (52%) of parents reported that they would be “very” or “somewhat” comfortable with an orthodontist administering the HPV vaccine to their children. Approximately a third (32%) of parents reported that they would be “very” or “somewhat” uncomfortable, and 16% were “neither uncomfortable nor comfortable” (Table 3).

Bivariable analysis results are shown in Table 3. The independent variables that were tested as possible predictors of comfort with orthodontists as HPV vaccinators are listed. According to this analysis, two variables (language of questionnaire and parents' highest education level) were statistically significant as to association with parents' comfort level of orthodontists being HPV vaccinators (*p* < .05). The language of the questionnaire analysis shows that parents taking the survey in Spanish were more comfortable with orthodontists as vaccinators than those taking the survey in English (*p* < .001). There was an inverse correlation between comfort level and parent's education level (*p*=.042). The lower the educational attainment level, the higher the comfort level with orthodontists as vaccinators.

Multivariate ordinal logistic regression was performed to identify any association between parent/child characteristics and parent's comfort level with orthodontists as HPV vaccinators (*p* < .05). The results are shown in Table 4 (n = 270 based on missing data for 5 patients). This analysis indicated a statistically significant association between language of the survey and comfort level; subjects taking the survey in Spanish were more than twice as likely (adjusted OR: 2.42, 95% CI: 1.24–4.72; *p* < .01) to be comfortable with an orthodontist administering the HPV vaccination compared to those taking it in English. Additionally, sex of the child had statistical significance to comfort level, with parents of male children being almost twice as likely (adjusted OR: 1.66, 95% CI: 1.01–2.73; *p* < .047) to be comfortable with orthodontists as HPV vaccinators than those with female children.

## 4. Discussion

These pilot data suggest that 52.4% of parents attending an inner city university orthodontic clinic are comfortable with orthodontists administering HPV vaccines to their children. Participants taking the survey in Spanish were found to be more comfortable with orthodontists administering HPV vaccines than those taking the survey in English. Parental education level and sex of the child also have statistically significant associations with comfort in orthodontists administering the vaccine.

Almost all of the participants who completed the questionnaire in Spanish identified themselves as Hispanic. This study showed that there was a statistically significant effect of the language used to complete the questionnaire on the parent's comfort level with orthodontists administering HPV vaccines to their child. In particular, those who completed the questionnaire in Spanish were more likely to be comfortable with orthodontists being HPV vaccinators than those who chose English. This finding was in agreement with other findings in literature that indicate that Hispanic and other minority adolescents are more likely to receive HPV vaccines than whites [27]. However, there are mixed findings in the literature regarding the effect of language on the HPV vaccine uptake or attitude toward the HPV vaccine. According to Ashing et al., Latinx who preferred English were found to be more knowledgeable about HPV vaccine and more likely to endorse its effectiveness and safety [28]. According to this same study, interventions are needed to improve the HPV vaccine uptake among Latinx populations that prefer Spanish over English by addressing the linguistic and socioecological differences [28]. In Galbraith et al.'s study focusing on vaccine acceptability among Latinx, they found that acceptability was associated with the benefits of preventing cancer with immunization and a recommendation by a healthcare provider [29]. They concluded that the linguistic barrier may contribute to lack of education or awareness of the benefits of HPV vaccines. The linguistic barrier may also contribute to a lack of healthcare provider's recommendations for HPV vaccination and, thus, a decrease in the HPV vaccine uptake among Hispanics that prefer Spanish over English. These findings conflict with the findings of our study, in which more than half of the subjects completed the questionnaire in Spanish and were more likely to be comfortable with orthodontists vaccinating their children with the HPV vaccine than those opting to take the survey in English.

The data in our study indicate that the level of parental education attainment has a negative association with parent comfort with orthodontist administering HPV vaccines to their children. Specifically, the higher the education level, the less comfortable the subjects with orthodontists administering a vaccine. This is in contrast to the Dean et al. and Lazalde et al. studies where they found no correlation between education attainment and acceptance of HPV vaccination by dentists [22. 23]. The reason for this is unknown but may be related to a smaller proportion of parents in our study attaining an education level higher than high school compared to the other studies.

Participants with male children were found to be more comfortable with orthodontists as HPV vaccine administrators than those with female children. This finding is concurrent with the Lazalde et al. study that also found that a parent with a female child had lower odds of being comfortable with their general dentist administering HPV vaccinations [22]. Prior studies have found that one of the main reasons why parents have not vaccinated their male children against HPV was a lack of provider recommendation [30]. Since our study found that parents of male children may be more comfortable with orthodontists giving the vaccine, orthodontists recommending and administering the vaccine could give a much needed boost to male vaccinations.

Not only could dental professionals help lower HPV infections by administering the vaccine, but they also could play a role in monitoring HPV mediated oral cancer progression. Salivary biomarkers can be used to detect HPV infections and HPV related oral squamous cell cancers. Dental professionals routinely incorporate oral cancer screenings into their patient exams. Since collection of a salivary sample is relatively easy, dental professionals could provide an important role in HPV related OSCC risk detection by incorporating salivary analysis as part of their oral cancer screening exams.

One limitation of this study involves the patient/parent population. The majority of patients who use dental services at the University are Hispanic. Since this is a convenience sample, it is not representative of the general population, but only of those in the area who access dental services at the university. Limitations also include the lack of data indicating how much cost is a factor in the decision to receive a vaccine. Studies have found that lack of access to healthcare via insurance results in lower acceptance of HPV vaccine uptake [19, 31]. The fact that most of the Hispanic patient population at the university have insurance coverage and, thus, perhaps better access to healthcare may have contributed to the positive association between survey language and Spanish participants' comfort with orthodontists as vaccinators. Another limitation is the lack of data on the child's vaccination status and the participant's views on vaccination in general. Anti-vaccinators, parents who are against administration of any vaccine to their child, would not consider the question we were attempting to ascertain in this study as they would answer negatively to all questions about vaccines and potential vaccinators. Thus, the responses to these questions may not reflect the preference of general population and may reflect the responses of only those who would not refuse to vaccinate their child.

The results of this study can be used to educate minority and Spanish-speaking populations regarding HPV vaccination administration by orthodontists and can be used to assess perceived comfort with administration of other vaccines. In response to the COVID-19 pandemic, the ADA House of Delegates passed a resolution in October of 2020 which acknowledges that dental professionals have the requisite knowledge and skills to administer critical vaccines. As a result, oral health professionals have been called upon by states to act as vaccine administrators of the multidose vaccine developed to combat COVID-19. Studies regarding patient comfort with oral health professionals administering vaccines will be a major step toward dentists becoming a permanent part of the current and future global vaccination effort.

Future research should assess orthodontists' readiness to assume a role as a vaccine administrator as well as ways of coordinating this effort with the patient's other healthcare providers. Additional research on other roles orthodontists can play in vaccine education/administration along with a more detailed analysis of the reasons that affect comfort level of parent's uptake of HPV vaccination for their children should also be investigated.

## Figures and Tables

**Table 1:** Parent and children demographic characteristics (*N* = 275).

Parent characteristics	*n*	(%)
Sex		
Male	54	(20.4)
Female	22	(79.6)
Age (in years)		
21–30 y	7	(2.5)
31–40 y	118	(42.9)
41–50 y	110	(40.0)
51–60 y	32	(11.6)
>60 y	8	(2.9)
Educational attainment		
Less than high school	75	(27.2)
High school graduate	105	(38.2)
Bachelor's degree	70	(25.5)
Master's degree	15	(5.5)
Doctoral degree	5	(1.8)
Missing^a^	5	(1.8)
Race/ethnicity		
Non-Hispanic whites	33	(12.0)
Non-Hispanic blacks	27	(9.8)
Hispanic	196	(71.3)
Others^b^	18	(6.5)
Missing^a^	1	(0.4)
Chosen language of survey		
English	123	(44.7)
Spanish	152	(55.3)
Relationship to child		
Father	51	(18.5)
Mother	216	(78.5)
Legal guardian	8	(3.0)
Insurance status		
No (self-pay)	230	(83.6)
Yes (Medicaid)	45	(16.4)
*Child characteristics*		

Sex		
Male	126	(45.8)
Female	149	(54.2)
Age		
11–12 y	87	(31.6)
13–15 y	148	(53.8)
16–17 y	40	(14.6)

^a^Did not answer the question. ^b^Other race includes parents who identified themselves as Asian, more than one race, or none of these.

**Table 2:** Parent comfort level with orthodontists as HPV vaccinators (*N* = 275).

Role of orthodontists	Level of parents' comfort									
	Very uncomfortable		Somewhat uncomfortable		Neither uncomfortable nor comfortable		Somewhat comfortable		Very comfortable	
	*n*	(%)	*n*	(%)	*n*	(%)	*n*	(%)	*n*	(%)
Child's orthodontist administering the HPV vaccine to the child	38	(13.8)	50	(18.2)	43	(15.6)	52	(18.9)	92	(33.5)
	Uncomfortable: *n* = 88, (32.0)						Comfortable: *n* = 144, (52.4)			

**Table 3:** Bivariable analysis (Kruskal–Wallis H test): parents' comfort with orthodontists administering HPV vaccinations to their adolescent children by parent and child characteristics (*N* = 275).

		Comfortable (*n* = 144, 52.4%)		Neither uncomfortable nor comfortable (*n* = 43, 15.6%)		Uncomfortable (*n* = 88, 32.0%)		*p* value
		*n*	(%)	*n*	(%)	*n*	(%)	
*Parent characteristics*								
Sex	Male	29	(20.1)	8	(18.6)	17	(19.3)	.972
	Female	115	(79.9)	35	(81.4)	71	(80.7)	
Age	21–30 y	3	(2.1)	2	(4.7)	2	(2.3)	.479
	31–40 y	61	(42.3)	22	(51.1)	35	(39.8)	
	41–50 y	57	(39.6)	12	(27.9)	41	(46.6)	
	51–60 y	18	(12.5)	7	(16.3)	7	(7.9)	
	>60 y	5	(3.5)	0	(0.0)	3	(3.4)	
Educational attainment	Less than high school	43	(29.9)	14	(32.6)	18	(20.5)	.042⁣^*∗*^
	High school graduate	57	(39.6)	16	(37.2)	32	(36.4)	
	Bachelor's degree	31	(21.5)	12	(27.9)	27	(30.7)	
	Master's degree	7	(4.9)	0	(0.0)	8	(9.1)	
	Doctoral degree	3	(2.1)	0	(0.0)	2	(2.3)	
Race/ethnicity	Non-Hispanic whites	16	(11.1)	4	(9.3)	13	(14.8)	.664
	Non-Hispanic blacks	13	(9.0)	4	(9.3)	10	(11.4)	
	Hispanic	103	(71.5)	35	(81.4)	58	(65.9)	
	Others	11	(8.3)	0	(0.0)	7	(7.9)	
Chosen language of survey	English	59	(41.0)	9	(20.9)	55	(62.5)	<.001⁣^*∗*^
	Spanish	85	(59.0)	34	(79.1)	33	(37.5)	
Relationship to child	Father	28	(19.4)	8	(18.6)	15	(17.0)	.926
	Mother	113	(78.5)	33	(76.7)	70	(79.6)	
	Legal guardian	3	(2.1)	2	(4.7)	3	(3.4)	
Insurance status	No	124	(86.1)	38	(88.4)	68	(77.3)	.140
	Yes	20	(13.9)	5	(11.6)	20	(22.7)	

*Child characteristics*								
Sex	Male	74	(51.4)	16	(37.2)	36	(40.9)	.141
	Female	70	(48.6)	27	(62.8)	52	(59.1)	
Age (in years)	11–12 y	46	(31.9)	16	(37.2)	25	(28.4)	.394
	13–15 y	73	(50.7)	24	(55.8)	51	(58.0)	
	16–17 y	25	(17.4)	3	(7.0)	12	(13.6)	

⁣^*∗*^*p* < 0.05.

**Table 4:** Multivariate ordinal logistic regression: association between parent and children characteristics against parents' comfort level in orthodontics administering HPV vaccination to their children (*N* = 270).

		Parents comfort leveluncomfortable (*n* = 87, 32.2%) > neither (n-42, 15.6%) > comfortable (*n* = 141, 52.2%)		
		aOR	(95%CI)	*p* value
Parent characteristics				
Sex	Female	Ref		
	Male	0.32	(0.02–4.64)	.400
Age	21–30 y	Ref		
	31–40 y	2.36	(0.54–10.38)	.256
	41–50 y	2.20	(0.49–9.83)	.302
	51–60 y	3.01	(0.61–14.76)	.175
	>60 y	2.73	(0.34–22.05)	.346
Educational attainment	Less than high school	Ref		
	High school graduate	0.93	(0.50–1.72)	.806
	Bachelor's degree	0.74	(0.36–1.56)	.430
	Master's degree	0.63	(0.17–2.34)	.491
	Doctoral degree	0.74	(0.10–5.33)	.766
Race/ethnicity	Non-Hispanic whites	Ref		
	Non-Hispanic blacks	0.82	(0.25–2.63)	.733
	Hispanic	0.50	(0.19–1.37)	.180
	Others	1.07	(0.29–3.94)	.918
Chosen language of Survey	English	Ref		
	Spanish	2.38	(1.22–4.65)	.01^∗^
Relationship to Child	Mother	Ref		
	Father	3.57	(0.23–56.78)	.367
	Legal guardian	0.53	(0.12–2.34)	.402
Insurance Status	No	Ref		
	Yes	0.622	(0.28–1.38)	.244
Child characteristics				
Sex	Female	Ref		
	Male	1.66	(1.01–2.73)	.046^∗^
Age (in years)	11–12 y	Ref		
	13–15 y	0.79	(0.46–1.36)	.389
	16–17 y	1.17	(0.52–2.65)	.709

⁣^*∗*^*p* < 0.05.

## Data Availability

The survey questionnaire used to support the findings of this study is included within the supplementary information files. The data (answers to the questionnaire in the form of an Excel spreadsheet) used to support the findings of this study are available from the corresponding author upon request.

## Supplementary Materials

Supplementary Materials The supplementary file includes the “Parent attitudes regarding orthodontists' role as potential administrators of human papilloma virus (HPV) vaccines.”
