# Correction to “Parent Attitudes regarding Orthodontists’ Role as Potential Administrators of Human Papilloma Virus (HPV) Vaccines”

**DOI:** 10.1155/ijod/9876870

**Published:** 2025-12-30

G. Lee, J. Begley, K. Ahluwalia, et al., “Parent Attitudes regarding Orthodontists’ Role as Potential Administrators of Human Papilloma Virus (HPV) Vaccines,” *International Journal of Dentistry* 2022 (2022): 6541532, https://doi.org/10.1155/2022/6541532.

In the article titled “Parent Attitudes regarding Orthodontists’ Role as Potential Administrators of Human Papilloma Virus (HPV) Vaccines,” there was an error in the *p*‐values in the Abstract and Section 3.2.

*p*  < 0.047 should read *p*  < 0.046.

We apologize for these errors.

