# Supplementary material for: Parent Attitudes regarding Orthodontists' Role as Potential Administrators of Human Papilloma Virus (HPV) Vaccines
Source: Int J Dent. 2022 Jun 6;2022:6541532. doi: 10.1155/2022/6541532 (PMC9192217; doi:10.1155/2022/6541532)
Supplement: Supplementary Materials — The supplementary file includes the “Parent attitudes regarding orthodontists' role as potential administrators of human papilloma virus (HPV) vaccines.” [file 6541532.f1.pdf]

We would like to ask a few questions about your child's oral health care.

During the past 12 months, how many times did your child see an orthodontist for routine treatment?

1. None
2. 1 time
3. 2 times or more

Does your child have an orthodontist where he/she goes regularly?

0. No
1. Yes

Some types of HPV can cause mouth and throat cancer. The HPV vaccine can protect against some of these types.

How comfortable would you be with receiving written information about the HPV vaccine from your child's orthodontist?

1. Very uncomfortable
2. Somewhat uncomfortable
3. Neither uncomfortable or comfortable
4. Somewhat comfortable
5. Very comfortable

How comfortable would you be with your child's orthodontist talking to you about the HPV vaccine?

1. Very uncomfortable
2. Somewhat uncomfortable
3. Neither uncomfortable or comfortable
4. Somewhat comfortable
5. Very comfortable

How comfortable would you be with your child's orthodontist talking to him/her about the HPV vaccine?

1. Very uncomfortable
2. Somewhat uncomfortable
3. Neither uncomfortable or comfortable
4. Somewhat comfortable
5. Very comfortable

How comfortable would you be with your child's orthodontist recommending that he/she get the HPV vaccine from his/her regular doctor?

1. Very uncomfortable
2. Somewhat uncomfortable
3. Neither uncomfortable or comfortable
4. Somewhat comfortable
5. Very comfortable

Has an orthodontist ever talked with you about HPV or the HPV vaccine?

0. No
1. Yes
2. I don't know

In some states, orthodontists who have completed special training are allowed to give vaccines.

Imagine you and your child decided to get him/her the HPV vaccine.
How comfortable would you be with him/her getting it from an orthodontist?

1. Very uncomfortable
2. Somewhat uncomfortable
3. Neither uncomfortable or comfortable
4. Somewhat comfortable
5. Very comfortable

What concerns would you have about your child getting the HPV vaccine at the orthodontist's office?

1. Insurance might not cover it
2. Staff might not be good at giving shots
3. Staff might not be able to deal with side effects
4. Staff might not be able to answer my questions
5. Orthodontists should not give vaccines
6. I would rather get it from my child's regular doctor
7. I want my child's regular doctor to keep track of his/her vaccines
8. I would have no concerns

What benefits would you see of your child getting the HPV vaccine at the orthodontist's office?

1. It would be convenient
2. I trust my child's orthodontist to give shots
3. Staff would be skilled at giving shots
4. My child would be comfortable getting it there
5. orthodontists are experts in oral health
6. I would see no benefits

As a reminder, the HPV vaccine requires 3 shots over six months.

If you and your child decided to get him/her the HPV vaccine, which of these options would you most prefer?

1. To get all of the shots from a doctor
2. To get all of the shots from an orthodontist
3. To get the first shot from a doctor and “follow-up” shots from an orthodontist

We would like to ask a few questions about you and your child.

What is the age of your child?

1. 11
2. 12
3. 13
4. 14
5. 15
6. 16
7. 17

What is the age of the person filling out this survey?

1. 21-30
2. 31-40
3. 41-50
4. 51-60
5. 61-70

What is the sex of your child?

1. Female
2. Male

What is the sex of the person filling out this survey?

1. Female
2. Male

What is the zip code of your residence? _______________

Columbia University IRB
IRB-AAAS3486 (Y01M00)
IRB Exemption Date: 04/18/2019

What is your relationship to the child?

1. Mother
2. Father
3. Legal guardian

What is your race/ethnicity?

1. White, non-Hispanic
2. Black, non-Hispanic
3. Hispanic
4. Asian, non-Hispanic
5. American Indian or Alaska Native, non-Hispanic
6. Native Hawaiian or Pacific Islander, non-Hispanic
7. More than one race
8. Other, non-Hispanic

What is your highest education level?

1. Below high school
2. High school graduate
3. Bachelor's degree
4. Master's degree
5. Doctoral degree

Thank you for participating in the survey.

Reference: Lazalde GE, Gilkey MB, Kornides ML, McRee AL. Parent perceptions of dentists' role in HPV vaccination. Vaccine. 2018 Jan 25;36(4):461-466.

Columbia University IRB
IRB-AAAS3486 (Y01M00)
IRB Exemption Date: 04/18/2019
